# Local Variations in CO and Particulate Air Pollution and Adverse Birth Outcomes in Los Angeles County, California, USA

**DOI:** 10.1289/ehp.7751

**Published:** 2005-05-10

**Authors:** Michelle Wilhelm, Beate Ritz

**Affiliations:** 1 Department of Epidemiology and; 2 Center for Occupational and Environmental Health, School of Public Health, University of California at Los Angeles, Los Angeles, California, USA

**Keywords:** air pollution, epidemiology, low birth weight, preterm birth

## Abstract

We extended our previous analyses of term low birth weight (LBW) and preterm birth to 1994–2000, a period of declining air pollution levels in the South Coast Air Basin. We speculated that the effects we observed previously for carbon monoxide, particulate matter < 10 μm in aero-dynamic diameter (PM_10_), and traffic density were attributable to toxins sorbed to primary exhaust particles. Focusing on CO, PM_10_, and particulate matter < 2.5 μm in aerodynamic diameter (PM_2.5_), we examined whether varying residential distances from monitoring stations affected risk estimates, because effect attenuation may result from local pollutant heterogeneity inadequately captured by ambient stations. We geocoded home locations, calculated the distance to the nearest air monitors, estimated exposure levels by pregnancy period, and performed logistic regression analyses for subjects living within 1–4 mi of a station. For women residing within a 1-mi distance, we observed a 27% increase in risk for high (≥ 75th percentile) first-trimester CO exposures and preterm birth and a 36% increase for high third-trimester pregnancy CO exposures and term LBW. For particles, we observed similar size effects during early and late pregnancy for both term LBW and preterm birth. In contrast, smaller or no effects were observed beyond a 1-mi distance of a residence from a station. Associations between CO and PM_10_ averaged over the whole pregnancy and term LBW were generally smaller than effects for early and late pregnancy. These new results for 1994–2000 generally confirm our previous observations for the period 1989–1993, again linking CO and particle exposures to term LBW and preterm birth. In addition, they confirm our suspicions about having to address local heterogeneity for these pollutants in Los Angeles.

Over the past few years, the number of reports linking outdoor air pollution to adverse birth outcomes including intrauterine growth retardation, preterm birth, and perinatal mortality increased considerably (Glinianaia et al. 2004; Maisonet et al. 2004). The fast expansion of this research worldwide was enabled by the existence of air monitoring stations and routinely collected birth certificate information in many populated urban areas. The studies conducted in many different locales and populations agree in one aspect: Outdoor air pollution seems to play some role in determining birth outcomes. Yet the differences in pollutants, outcomes, and pregnancy periods studied make causational interpretations of the observed associations a subject of ongoing debate. Although local monitoring resources and major emission sources may determine choices for pollutants studied, it is time to use all available data as comprehensively as possible and to consider asking some new questions to further expand and eventually integrate our knowledge base.

Our previous work focused on the South Coast Air Basin (SoCAB) of Southern California and examined adverse birth effects due to air pollution in infants born between 1989 and 1993. Exposure assessment was based on measurements taken at air monitoring stations located throughout the basin. We observed positive associations between average carbon monoxide concentrations during the third trimester of pregnancy and term low birth weight (LBW) (Ritz and Yu 1999) and between concentrations of CO and particulate matter < 10 μm in aerodynamic diameter (PM_10_) 6 weeks before birth and prematurity (Ritz et al. 2000). We also reported a dose–response relationship between CO concentrations during the second month of pregnancy and cardiac ventricular septal defects and between second-month ozone concentrations and aortic/pulmonary artery and valve anomalies and conotruncal defects (Ritz et al. 2002).

Here we not only extend our previous analyses of term LBW and preterm birth to a more recent period during which air pollution levels in the SoCAB generally declined (1994–2000), but also examine issues that previously could not be addressed. We reported that proximity to traffic sources were related to these birth outcomes, suggesting that smaller primary exhaust particles may play a role for the effects we observed in the SoCAB (Wilhelm and Ritz 2003). Ambient monitoring stations, however, may not adequately capture the effects of primary exhaust pollutants that are more heterogeneously distributed throughout neighborhoods such that exposure depends on proximity to sources. Recently we obtained two new data sources: electronic birth address data for Los Angeles (LA) County and fine particle [particulate matter < 2.5 μm in aerodynamic diameter (PM_2.5_)] monitoring data collected in the SoCAB since 1999. The address data allowed us to examine the potential for and magnitude of exposure misclassification resulting from local heterogeneity in pollutant exposures. To do so, we relied on residential distance to monitoring stations because localized exposure might be captured more accurately for residences in closer proximity to a monitoring station. It has been argued that smaller particles are of most relevance for human health (Englert 2004; Ibald-Mulli et al. 2002). Based on emission inventories, most fine and ultra-fine (PM_<0.1_) particles found in the urban atmosphere derive from engine combustion (Hitchins et al. 2000; Schauer et al. 1996; Shi et al. 1999; Zhu et al. 2002a), and most particles emitted directly in vehicle exhaust are in the ultrafine size range of 20–130 nm for diesel engines and 20–60 nm for gasoline engines (Morawska et al. 1999; Shi et al. 2001). Recent dosimetry studies indicate the total deposition fraction of ultrafine particles increases as particle size decreases, with the greatest fractional deposition in the deep lung occurring between 5 nm and 100 nm (Jaques and Kim 2000; Yeh et al. 1997). Unlike larger fine particles, ultrafines seem to escape phagocytosis by alveolar macrophages and translocate to extrapulmonary organs (Oberdörster and Utell 2002); thus, they may be able to transfer potentially toxic compounds sorbed to these particles—such as polycyclic aromatic hydrocarbons (PAHs)—to the fetus and the placenta. It has been suggested that these compounds may interfere with placental development and subsequent nutrient and oxygen delivery to the fetus (Dejmek et al. 1999, 2000).

Topinka et al. (1997) reported PAH–DNA adduct levels in placentas from nonsmoking women living in a polluted district in the Czech Republic to be significantly greater than those in placentas of women living in an agricultural area with lower air pollution levels. Perera et al. (1998) reported decreased birth weights, lengths, and head circumferences in Polish newborns with elevated PAH–DNA adduct levels in cord blood leukocytes, and in a more recent study (Perera et al. 2003) conducted in New York City, they observed lower birth weights and head circumferences in babies born to African-American women exposed to high PAH levels during pregnancy. For our large population-based study, neither ultrafine particle nor placental PAH–DNA adduct measurements were available; instead, we relied on PM_10_, PM_2.5_, and CO as exposure proxies. CO is released directly in motor vehicle exhaust and does not react readily in the atmosphere to form other compounds. Also, decreases in CO concentrations as one moves farther away from traffic sources in LA correlate almost perfectly with decreases in ultrafine particle number counts and black smoke concentrations (Zhu et al. 2002a, 2002b). However, significant amounts of PM_2.5_ are created secondarily through atmospheric reactions depending on season and location in the LA Basin (Kim et al. 2002). Thus, although the new PM_2.5_ measures allow us to examine the contribution of fine particles to the observed effects on adverse birth outcomes, they cannot be easily interpreted as a primary exhaust proxy, and CO may still be the better indicator/proxy of primary exhaust toxins’ contributions.

## Materials and Methods

### Subjects.

We used birth certificates, provided by the California Department of Health Services, to identify study subjects and to determine their gestational age, birth weight, and information on covariates included in our analyses. To allow comparisons with our previous results for the period 1989–1993 (Ritz and Yu 1999; Ritz et al. 2000), we performed a ZIP-code–level analysis in which we selected all births during 1994–2000 to mothers who resided in a ZIP code whose area fell at least 60% within a 2-mi radius of a monitoring station (31 SoCAB ZIP codes met this criterion in 1994–2000, resulting in a total of 146,972 births). The 2-mi criterion is based on the assumption that stationary air monitors may most accurately reflect air pollution exposures within a small area surrounding stations, especially for pollutants with concentrations that vary spatially according to local sources, such as CO.

In a second, address-level analysis, we identified all 1994–2000 births to women living in ZIP codes located within a broader 5-mi radius of a monitoring station in LA County (any portion of the ZIP code). We obtained electronic address data from the LA County Department of Health and linked these to the state-level data based on unique identifiers (local file number, date of birth, and ZIP code) for 930,681 (93.6%) of the 994,832 births in these ZIP codes. We geocoded these home locations using ArcView GIS software (version 3.2) and StreetMap (both from Environmental Systems Research Institute, Redlands, CA). After correcting addresses that could not be geocoded during the first round of automated processing in ArcView (*n* = 87,647) with ZP4 software (August 2002 data release; Semaphore Corporation, Aptos, CA), we were able to map 47,583 additional subjects based on corrected addresses. Thus, overall we mapped 840,472 subject homes (90.3% of homes that could be address matched); unsuccessful mapping was due to address errors or an inability to match recorded house numbers to street segments in the StreetMap.

Calculating the distance from each home to the nearest air monitoring station, we found that 518,254 subjects resided within 4 mi of a station. Of the 146,972 (2-mi ZIP-code approach) and 518,254 (4-mi address approach) subjects, 141,475 and 498,235 records, respectively, provided gestational age and birth weight data. We excluded infants with birth weights < 500 g (*n* = 139 for ZIP-code and 511 for address analyses, respectively) or ≥ 5,000 g (*n* = 265 and 891) and births for which gestational age was likely misreported [delivery occurred < 90 days (*n* = 56 and 213) or ≥ 320 days gestation (*n* = 1,639 and 6,086)]. We also restricted our sample to singleton births (excluding 3,242 and 11,365 multiple births, respectively). Finally, some subjects were excluded because of insufficient monitoring data available during the pregnancy periods of interest: < 30 or 10 days of measurements available for CO, NO_2_, and O_3_ during a given trimester or month/6 week period of pregnancy, respectively; < 5 or 2 days of measurements available for PM_10_ during a given trimester or month/6-week period of pregnancy, respectively; or < 10 or 4 days of measurements available for PM_2.5_ during a given trimester or month/6-week period of pregnancy, respectively. In our adjusted analyses, study subjects may also have been excluded because of missing data for individual-level covariates such as maternal age, infant sex, maternal race, prenatal care information, and maternal education; final sample sizes are reported along with the results.

The outcomes of interest were term LBW (< 2,500 g at ≥ 37 completed weeks gestation) and vaginal birth < 37 completed weeks gestation; for analyses of preterm birth, we excluded births delivered by cesarean section because we previously found no evidence that these were related to increased air pollution levels before delivery (Ritz et al. 2000). Outcomes were analyzed as dichotomous variables, such that term LBW or preterm babies were compared with all other infants who were born at term and weighed ≥ 2,500 g at birth. We generated odds ratio (OR) or risk ratio (RR) estimates for term LBW and preterm birth. This research was approved by the University of California at Los Angeles Office for Protection of Research Subjects and the California State Committee for the Protection of Human Subjects.

### Exposure assessment.

Maternal exposure to air pollution during various pregnancy periods was estimated based on air monitoring data for CO, nitrogen dioxide, O_3_, PM_10_, and PM_2.5_ collected by the South Coast Air Quality Management District (SCAQMD) at 16 (2-mi ZIP-code approach) and 12 stations (4-mi address approach) between 1994 and 2000. For the ZIP-code–level analysis, O_3_ measurements were available at all 16 stations, CO and NO_2_ measurements were available at 15 stations, PM_10_ measurements at 8 stations, and PM_2.5_ measurements at 9 stations in 1999–2000. For the address-level analysis (focused on LA County), CO and O_3_ measurements were available at all 12 stations, and NO_2_, PM_10_, and PM_2.5_ measurements were available at 11, 6, and 8 stations, respectively. Based on the birth date and gestational age reported on the birth certificate, we calculated the start and end dates of various pregnancy periods for each subject (entire pregnancy, trimesters and months of pregnancy, and 6 weeks before birth) and averaged air pollution concentrations measured at the assigned station over these periods. The averages were based on hourly measurements for the gaseous pollutants (CO, NO_2_, and O_3_); 24-hr average measurements taken every 6 and 3 days were available for PM_10_ and PM_2.5_, respectively. We evaluated associations between risk of term LBW and average air pollution exposures during each trimester and over the entire pregnancy period. For preterm birth, we focused on exposures during the first month of pregnancy, the first and second trimesters of pregnancy, and 6 weeks before birth.

### Statistical methods.

The association of air pollution with term LBW and preterm birth was evaluated using logistic regression analyses. We evaluated air pollution exposures as continuous measures and grouped them into categories according to their distribution in the total population (< 25th, 25th to < 75th, and ≥ 75th percentiles). Exposure to levels below the 25th percentile was used as the referent category for each pollutant.

We adjusted for several known risk factors for LBW and preterm birth that could potentially confound the relationship between adverse birth outcomes and air pollution. For all outcomes, we adjusted for maternal age (< 20, 20–29, 30–34, 35–39, ≥ 40 years), maternal race (African American, white, Hispanic, Asian, other races), maternal education (< 9, 9–11, 12, 13–15, ≥ 16 years), parity (first birth vs. second or subsequent birth), interval since the previous live birth (≤ 12 months vs. > 12 months), level of prenatal care (none, during first trimester, after first trimester), infant sex, previous LBW or pre-term infant (one or more vs. none), and birth season (Table 1). For birth weight, we also adjusted for gestational age (measured in weeks), entering a linear and quadratic term into the model to capture the leveling off of the slope for weight gain during the last weeks of pregnancy (Ritz and Yu 1999). Risk factors for LBW and preterm birth that are not registered on California birth certificates include maternal active and passive smoking, maternal weight and height, pregnancy weight gain, birth weight of mother, and marital status. We performed separate analyses for subjects living near stations that monitored CO but not PM_10_ versus those that provided measures for both CO and PM_10_.

## Results

In Tables 1 and 2, we present mean birth weights, gestational ages, and the incidences of term LBW and preterm birth by known risk factors and by percentiles of air pollution exposure during various pregnancy periods. We found the highest incidence of term LBW and preterm birth among mothers who lacked prenatal care, were of African-American race, experienced previous low weight or preterm births, and were younger (< 20 years) or older (≥ 40 years) at delivery. In contrast, the incidence of term LBW and preterm birth was lower among women with higher educational levels, higher order parity, and at least 12 months since the previous live birth. In female infants, the incidence of term LBW was higher but the incidence of preterm birth was lower than in male infants, and more preterm babies were born during the winter months. Incidences based on the address-level cohort were similar.

Table 3 presents pollutant means and correlations based on the ZIP-code–level analyses; correlations based on the address-level analyses were very similar. Pregnancy averages for CO, NO_2_, and PM_2.5_ were strongly positively correlated with each other and inversely correlated with O_3_. In the SoCAB, this is due to well known seasonal and geographic patterns for these pollutants. PM_10_ averages were moderately correlated with PM_2.5_, NO_2_, and CO.

## Term LBW

### CO effects.

**Figure 1:**
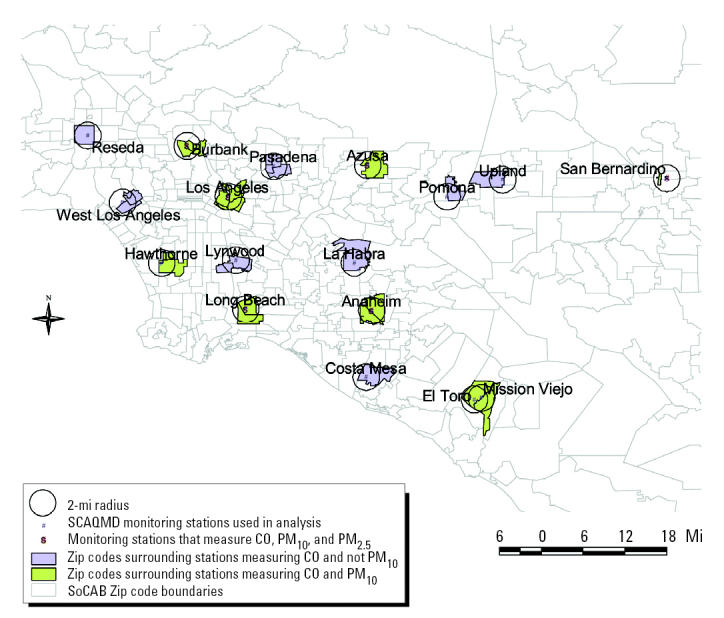
Location of SoCAB monitoring stations measuring CO and PM_10_ and CO only: ZIP-code–level analysis.

**Figure 2:**
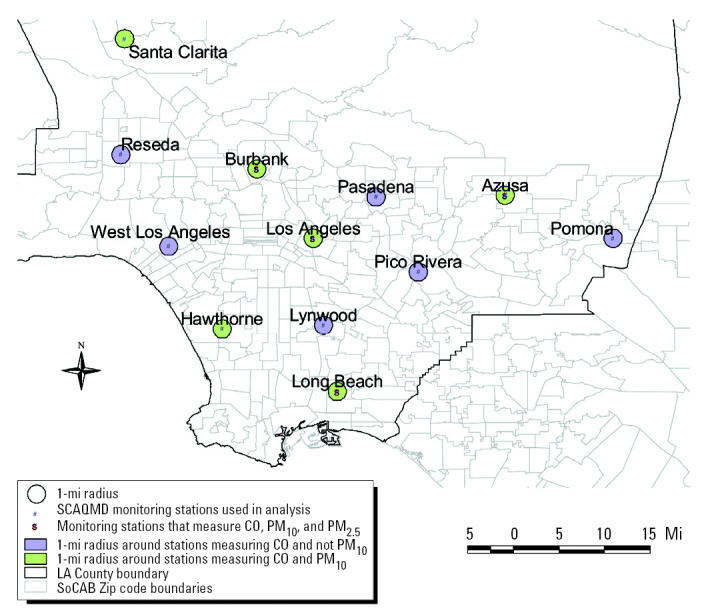
Location of LA County monitoring stations measuring CO and PM_10_ and CO only: address-level analysis.

We observed a 12% increase in risk of term LBW per 1-ppm increase in third-trimester CO in ZIP-code–level analyses and a 10% increase for women living within 1 mi of a station based on single-pollutant models (Table 4). Beyond 1 mi of a station, the estimated effect sizes were smaller (~ 5% increase per 1 ppm CO). Adding NO_2_ and O_3_ average third-trimester concentrations to our models did not change the positive associations observed for CO, but adding PM_10_ had opposite effects at the ZIP-code and address level. The point estimates for CO were close to 1 in PM_10_-adjusted ZIP-code analyses, whereas for women living within 1 mi of a station the effects for CO persisted after adjustment for PM_10_. However, because fewer stations measure PM_10_, adding these averages reduced our sample size for each model considerably and resulted in a loss of precision for the 1-mi radius analyses. We performed analyses separately for stations measuring both pollutants versus CO only [referred to below as CO-only stations (Figures 1 and 2); results not shown in tables] and found that the effect for CO appeared isolated to women residing near stations measuring CO but not PM_10_. In fact, in ZIP-code–level analyses we observed an 18% [OR for the single-pollutant model (OR_single_) = 1.18; 95% confidence interval (CI), 1.09–1.29] increase in term LBW risk per 1-ppm increase in third-trimester CO for women residing near monitoring stations that measured CO but not PM_10_, whereas for residents living around stations measuring both pollutants, effect estimates were close to 1 in single- and multipollutant models (per 1-ppm increase: OR_single_ = 0.99; 95% CI, 0.89–1.09; OR_multi_ = 0.99; 95% CI, 0.85–1.15). For women living within 1 mi of a station, our results also suggested some increases for CO at CO-only stations (per 1-ppm increase: OR_single_ = 1.07; 95% CI, 0.93–1.24), whereas at stations also measuring PM_10_, CO was associated with term LBW only after adjustment for particles (per 1-ppm increase: OR_multi_ = 1.21; 95% CI, 0.85–1.74), suggesting confounding of CO associations by PM_10_ at these stations.

Effect estimates for CO concentrations averaged over the entire pregnancy period and term LBW were similar to the third-trimester results at the ZIP-code–level (per 1-ppm increase: OR_single_ = 1.12; 95% CI, 1.04–1.20; adjusting for PM_10_: OR_multi_ = 0.93; 95% CI, 0.76–1.13) and for women residing within 1 mi of a station (per 1-ppm increase: OR_single_ = 1.05; 95% CI, 0.91–1.22; adjusting for PM_10_: OR_multi_ = 1.00; 95% CI, 0.62–1.59). Again, the associations seemed isolated to women living near stations measuring CO only (per 1-ppm increase: OR_single_ = 1.09; 95% CI, 0.91–1.30) versus those living within 1 mi of stations measuring both pollutants (per 1-ppm increase: OR_multi_ = 1.00; 95% CI, 0.62–1.59), yet these estimates suffered reduced precision because of the much smaller sample size within the 1-mi distance.

### Particle effects.

Unlike the ZIP-code–level analysis that provided no evidence for an effect of PM_10_ concentrations on term LBW risk, a 48% increase in risk was observed for women with third-trimester PM_10_ averages of ≥ 44.0 μg/m^3^ and residing within 1 mi of an LA County station in a single-pollutant model (Table 4). The effect estimates for PM_10_ slightly increased to 58% when adding other pollutants to the model, but 95% CIs widened because of the reduction in sample size. Relatively strong associations were also observed for women residing within 1 mi of a monitoring station in multipollutant models for the third-trimester (per 10 μg/m^3^: OR_multi_ = 1.36; 95% CI, 1.12–1.65) and entire pregnancy period (per 10 μg/m^3^: OR_multi_ = 1.24; 95% CI, 0.91–1.70). Although CIs for percentile-based estimators were wide, the continuous variables suggested an exposure–response pattern. No associations were observed when the distance between subject homes and monitoring locations was greater than 1 mi. The sample size for PM_2.5_—only available for the years 1999–2000—was too limited and resulted in CIs too wide to derive conclusive results for this outcome.

### Other pollutants and pregnancy periods.

No associations were observed between first- and second-trimester CO and PM_10_ concentrations and term LBW based on ZIP-code–level analyses or for first- and second-trimester PM_10_ concentrations based on address-level analyses. However, address-level analyses suggested effects for first-trimester CO for women living within 1 mi of a station, but only after adjusting for NO_2_ and O_3_ [per 1 ppm: OR adjusted for gaseous pollutants (OR_adjusted_) = 1.07; 95% CI, 0.90–1.28; no association when PM_10_ was added to the model]. Similarly, associations between second-trimester CO and term LBW were suggested for women living within 1 mi of a station (per 1 ppm: OR_adjusted_ = 1.09; 95% CI, 0.99–1.19).

After adjusting for CO and/or PM_10_, we did not observe associations between NO_2_ and O_3_ and term LBW in any of our models.

## Preterm birth

### CO effects.

Focusing first on early pregnancy, in the ZIP-code and address-level analyses we observed a 4–8% increase in risk of preterm birth per 1-ppm increase in first-trimester CO that persisted when adjusting for gaseous pollutants; however, point estimates were close to 1 after adjustment for PM_10_ (Table 5). Stratifying on station type revealed that the associations again applied only to women who lived close to stations measuring CO and not PM_10_ (CO ≥ 2.2 ppm: RR_adjusted_ = 1.24; 95% CI, 1.00–1.54) and not to women living within 1 mi of stations monitoring both pollutants (CO ≥ 1.9 ppm: RR_multi_ = 1.03; 95% CI, 0.78–1.36). Results based on a shorter averaging period to reflect time of fetal implantation into the uterus—that is, the first month of pregnancy—were similar to those for first-trimester exposures. Furthermore, a small risk increase suggested for second-trimester CO exposures for women residing within 1 mi of a station disappeared when adjusting for PM_10_ exposures.

Examining influences of pollutant exposures at the end of pregnancy, we observed a 4–9% increase in the risk of preterm birth when average CO concentrations 6 weeks before birth were ≥ 1.9 ppm based on ZIP-code–level analyses (Table 5). Again, all associations were reduced and close to 1 when we adjusted for PM_10_, and estimated effects were limited to women residing near stations measuring CO and not PM_10_. In ZIP-code–level analyses, we estimated a 21% increase in risk for women residing near CO-only stations when average CO concentrations 6 weeks before birth were ≥ 2.0 ppm (RR_adjusted_ = 1.21; 95% CI, 1.06–1.38), whereas the estimate was close to 1 (CO ≥ 1.8 ppm: RR_multi_ = 0.94; 95% CI, 0.84–1.05) for women residing near stations measuring both pollutants. At CO-only stations, the effect was stronger and more consistent in address-level analyses as well: We observed a 26–30% increase in risk of preterm birth for women residing within 1–2 mi of a station (CO ≥ 2.1 ppm and residence within 1 mi: RR_adjusted_ = 1.26; 95% CI, 1.03–1.55; CO ≥ 2.1 ppm and residence within 1–2 mi: RR_adjusted_ = 1.30; 95% CI, 1.15–1.48), whereas at stations measuring both pollutants the CO point estimates were close to 1 (CO ≥ 1.8 ppm and residence within 1 mi: RR_multi_ = 0.85; 95% CI, 0.62–1.15; CO ≥ 1.8 ppm and residence within 1–2 mi: RR_multi_ = 0.97; 95% CI, 0.84–1.11).

### Particle effects.

We did not observe a risk increase for first-trimester PM_10_ exposures and preterm birth based on the ZIP-code–level analyses. Yet women in the highest exposure quartile and residing within 1 mi of a station experienced a 17% increased risk during early pregnancy (PM_10_ ≥ 51.2 μg/m^3^: RR_multi_ = 1.17; 95% CI, 0.92–1.50). This effect decreased with increasing distance from a station, especially after 2 mi (Table 5). Negative effects were seen for PM_2.5_ in single-pollutant models for the first trimester, but these reversed in multipollutant models (per 10 μg/m^3^ PM_2.5_ for the 1–2 mi radius: RR_multi_ = 1.18; 95% CI, 0.84–1.65). Results based on first month average concentrations for both pollutants were similar to those observed for first-trimester concentrations.

We also did not observe associations between average PM_10_ concentrations 6 weeks before delivery and risk of preterm birth based on the ZIP-code–level analyses. For women residing within 1 mi of a station, our models suggested that PM_10_ exposures 6 weeks before birth have effects (17% increased risk for women in the highest exposure quartile), although our analyses were imprecise because of small sample sizes (Table 5).

Elevated PM_2.5_ levels 6 weeks before birth resulted in a 19% increase in risk of preterm birth (PM_2.5_ ≥ 24.3 μg/m^3^: RR_single_ = 1.19; 95% CI, 1.02–1.40) based on the ZIP-code–level analysis, yet this estimate was reduced to 12% in a multipollutant model (PM_2.5_ ≥ 24.6 μg/m^3^: RR_multi_ = 1.12; 95% CI, 0.82–1.52) and was rather imprecise. Our continuous exposure measure suggested that the risk of preterm birth increased by 12% per 10-μg/m^3^ increase in PM_2.5_ averaged over 6 weeks before birth (RR_single_ = 1.10; 95% CI, 1.00–1.21; RR_multi_ = 1.12; 95% CI, 0.90–1.40). Point estimates were stronger for PM_2.5_ exposures 6 weeks before birth for women living within 1 mi of a station, especially in multiple-pollutant models; yet again due to relatively small sample sizes, the 95% CIs were wide, especially when adjusting for all other pollutants.

### Other pollutants and pregnancy periods.

We did not observe associations between first- and second-trimester NO_2_ concentrations and risk of preterm birth. We also observed no effects for second-trimester exposures to PM_10_ and PM_2.5_. When limiting the exposure period to the first month of pregnancy, O_3_ results for a model containing all pollutants showed strongly increased risks for preterm birth (per 1-pphm increase: RR = 1.23; 95% CI, 1.06–1.42; O_3_ ≥ 1.42 and < 2.97 pphm: RR = 1.45; 95% CI, 1.16–1.80; O_3_ ≥ 2.97 pphm: RR = 1.74; 95% CI, 1.31–2.32, based on the ZIP-code–level cohort); results for first-trimester exposures were similar but slightly smaller. Also, we observed a positive association between second-trimester O_3_ concentrations and risk of preterm birth, but only after including all pollutants in the model (per 1-pphm increase: RR = 1.38; 95% CI, 1.14–1.66). In general, models containing all pollutants (i.e., CO, NO_2_, O_3_, PM_10_, and PM_2.5_) were unstable because of collinearity between pollutant concentrations and the small sample size when including only 2 years of data for PM_2.5_. We observed no effects for NO_2_ and O_3_ concentrations 6 weeks before birth.

## Discussion

Our new results for 1994–2000 births generally confirm our previous observations for the period 1989–1993, again linking air pollution—specifically, CO and particles—to term LBW and preterm birth in the SoCAB and also confirmed our suspicions about the importance of addressing local heterogeneity in concentrations of pollutants from traffic sources.

Specifically, our ZIP-code–level analyses provided renewed evidence for an exposure–response relation between third-trimester CO concentrations and term LBW (Table 4), yet we observed the greatest effects for women living within 1 mi of a monitoring station (29–36% increased risk for the highest exposure quartile), and effect estimates clearly diminished with increasing distance between homes and stations. In accordance with our earlier results, ZIP-code–based analyses again showed no association between PM_10_ and term LBW. However, for women residing within 1 mi of a PM_10_ station, we estimated a relatively large 48–58% increase in term LBW risk for the highest third-trimester exposure quartile, and an exposure–response pattern was suggested. Unfortunately, sample sizes for the more recently established PM_2.5_ monitoring stations were too small, rendering our analyses for term LBW and PM_2.5_ uninformative. Thus, we cannot determine whether effects are related to fine or coarse particles or both.

In Western societies, birth weight is generally determined by factors affecting pregnancy after the 28th week of gestation (Kline et al. 1989). However, several researchers have hypothesized that exposure to particles and/or PAHs sorbed to particle surfaces may directly modulate the proliferation of the trophoblast because of reactions between these pollutants and receptors for placental growth factors (Dejmek et al. 2000, 1999; Perera et al. 1998), and this has also been borne out in some experimental studies (Guyda 1991; Zhang et al. 1995). Such reactions may interfere with fetoplacental exchange of oxygen and nutrients and subsequently impair fetal growth (Dejmek et al. 2000). Although previously we focused on third-trimester exposures for term LBW (Ritz and Yu 1999)—the period of pregnancy during which most fetal weight gain occurs—here we also examined effects for other trimesters and for exposures averaged over the entire pregnancy period, allowing comparisons with other studies. Our address-level analyses suggested effects for first- and second-trimester CO concentrations for women living within 1 mi of a monitoring station, but point estimates were lower than those for third-trimester exposures, and CIs were wide. Clearer effects emerged when averaging CO exposures over the entire pregnancy, yet the effect sizes were somewhat smaller than for third-trimester exposures only. Similarly, effects were suggested for PM_10_ averaged over the entire pregnancy period and term LBW risk; again, these estimates were smaller than those based on third-trimester exposures, and CIs were wide and included null values. Thus, our present results suggest that not only the third trimester but also the entire pregnancy period may influence term LBW at least for CO—that is, that the accumulation of exposure throughout pregnancy may affect fetal growth possibly in addition to peak exposures during especially vulnerable periods. Recently, a chronic/cumulative effect for smoking throughout pregnancy on perinatal mortality has also been suggested with risk increasing from early- to late-pregnancy exposures (Platt et al. 2004).

The existing literature on air pollution and adverse birth outcomes is difficult to synthesize because of differences in fetal growth and outcome measures, exposure periods, and pollutants evaluated in each study, and we concentrate here on those studies that can be compared with our own results. An early study reported that pregnancies in Beijing, China, were at increased risk of term LBW when average third-trimester concentrations of sulfur dioxide and total suspended particles (TSP) were high (per 100-μg/m^3^ increase in SO_2_: OR = 1.11; 95% CI, 1.06–1.16; per 100-μg/m^3^ increase in TSP: OR = 1.10; 95% CI, 1.05–1.14) (Wang et al. 1997). The study lacked measurements for CO and other pollutants possibly correlated with SO_2_ and TSP, and the main source of air pollution in Beijing at the time was residential use of coal stoves. Thus, generalizations to other urban areas more affected by transportation sources, such as southern California, may be limited, although the results implicated particle exposures during the third trimester, similar to our own study. More comparable with southern California may be the following studies conducted in the United States and other industrialized nations. A study of six northeastern U.S. cities found associations between third-trimester CO and term LBW (Maisonet et al. 2001), and a study of births in Washoe County, Nevada, estimated a mean birth weight reduction of 11 g (95% CI, 2.3–19.8 g) per 10-μg/m^3^ increase in PM_10_ during the third trimester (Chen et al. 2002); however, the latter study lacked statistical power when examining term LBW. Another U.S.-based study reported increased risks of very LBW (infants < 1,500 g) and term LBW for women residing in New Jersey census tracts with high polycyclic organic matter (POM) concentrations (PAHs comprise a major portion of POMs) (Vassilev et al. 2001a). These authors relied on modeled POM concentrations from the U.S. Environmental Protection Agency Cumulative Exposure Project that only allowed them to derive annual average concentrations, precluding the examination of exposure influences on specific pregnancy periods.

In Seoul, South Korea, first-trimester concentrations of CO, TSP, NO_2_, and SO_2_ increased the risk of term LBW, yet no associations were observed for third-trimester exposures (Ha et al. 2001). However, a follow-up study extending this Korean birth cohort by 2 years reported positive associations between first-trimester CO and, in addition, second-trimester CO, PM_10_, SO_2_, and NO_2_ concentrations and term LBW risk (Lee et al. 2003). Corroborating our new results for effect of exposure on term LBW throughout pregnancy, Lee et al. (2003) also reported positive odd ratios for each of the four pollutants averaged over the entire pregnancy.

Studies using small for gestational age (SGA) as an end point reported effects for first-trimester exposures to carcinogenic PAHs, PM_10_, and PM_2.5_ in the Czech Republic (Dejmek et al. 1999, 2000) and for first-month SO_2_, NO_2_, and CO exposures and first-trimester SO_2_ and CO exposures in Vancouver, Canada (Liu et al. 2003). The New Jersey study (Vassilev et al. 2001b) also reported increased SGA risk with elevated annual average POM concentrations. Studies focusing on LBW while adjusting for gestational age reported effects for early pregnancy exposures. A Czech study of LBW conducted by Bobak (2000) observed effects for first-trimester SO_2_ and TSP; how-ever, low gestational age accounted for this relation. The Vancouver study reported effects for first-month SO_2_ exposures and LBW risk similar to what they reported for SGA (Liu et al. 2003). Finally, some studies treated birth weight as a continuous outcome. Estimating birth weight reductions, Gouveia et al. (2004) reported inverse relations between first-trimester CO and PM_10_ concentrations and term birth weight for women in São Paulo, Brazil, adjusting for gestational age; however, they did not observe consistent relationships between term LBW and pollutant exposures in any specific trimester of pregnancy. A Taiwanese study also observed birth weight reductions in women exposed to higher first-trimester concentrations of SO_2_ and PM_10_, the only pollutants with measurements available (Yang et al. 2003). High prenatal exposures to PAHs were associated with lower birth weights and smaller head circumferences in African-American women living in New York City (Perera et al. 2003). Personal PAH samples during a 48-hr period in the third trimester were collected; thus, it is unclear whether these measurements represent exposures only during the third trimester or during all of pregnancy.

Concordance with our previous results was also observed for preterm birth: New ZIP-code–level analyses suggested small risk increases for CO exposures during early pregnancy (6% increase for the highest first-trimester exposure quartile) and late pregnancy (9% increase for the highest 6 weeks before birth exposure quartile). Again, our address-level analyses produced much larger CO effect estimates for women residing within 1–2 mi of a station compared with those living farther away. We observed no association between PM_10_ and risk of preterm birth in ZIP-code–level analyses, but a 20% increase in risk was suggested for women residing within 1 mi of a station when average first-trimester PM_10_ concentrations were ≥ 45.1 μg/m^3^; a 17% increase in risk was suggested for women residing within 1 mi of a station when average PM_10_ concentrations 6 weeks before birth were ≥ 44.8 μg/m^3^, yet our estimates were imprecise. An effect for exposures during the last 6 weeks before birth but not the first trimester was also observed for fine particles (< 2.5 μm): ZIP-code–level analyses revealed a 19% increase in risk of preterm birth for women with PM_2.5_ levels ≥ 24.7 μg/m^3^, and further address-level analyses suggested the strongest PM_2.5_ effects for women residing within 1 mi of a station, especially when controlling for all other pollutants.

The literature evaluating preterm birth as an outcome is less prolific than the literature on growth retardation. Similar to our earlier analysis (Ritz et al. 2000), we observed the strongest associations between air pollution and preterm birth for CO and PM_10_ in early pregnancy (first trimester) and late pregnancy (6 weeks before birth); it also appears that PM_2.5_ exposures in late pregnancy may be important. The Chinese study also reported a late pregnancy effect for air pollution in Beijing: Short-term increases in SO_2_ and TSP concentrations 7–10 days before birth increased the risk of preterm birth (Xu et al. 1995). The Vancouver study reported that SO_2_ and CO increases during the last month of pregnancy increased prematurity risk (Liu et al. 2003). Others reported effects on preterm birth for first-, second-, and third-trimester NO_2_ concentrations (Maroziene and Grazuleviciene 2002), first-trimester SO_2_ and TSP concentrations (Bobak 2000), annual average POM concentrations (Vassilev et al. 2001b), and an air pollution exposure index that combined annual average measures of five criteria pollutants (CO, NO_2_, O_3_, PM_10_, and SO_2_) (Woodruff et al. 2003). These data suggest that some component of urban air pollution (and it may not necessarily be a routinely measured component) seems to be acting in either early pregnancy or late pregnancy, or both, to increase susceptibility and/or trigger preterm birth. The biologic pathways for such triggering events in late pregnancy are to date unknown but may include disturbances of the pituitary–adrenocortico–placental system or uterine blood flow, and/or maternal infections initiating premature contractions and/or premature rupture of membranes. Toxicologic data may help answer these questions. Several studies including our own suggest, however, that the risk due to air pollution is greatest for exposures experienced in the first trimester. Hobel et al. (1999) reported that patients who delivered preterm had elevated plasma levels of adrenocorticotropic hormone at all gestational ages and elevated cortisol levels were observed already at 18–20 weeks’ gestation, suggesting that factors involved in the causation of pre-term birth may exert their influence earlier in gestation. Wadhwa et al. (2001) proposed that chronic rather than acute stressors or defined stress events need to be considered in advancing the understanding of risk factors for pre-term deliveries.

In general, we observed stronger associations for CO and term LBW and preterm birth when restricting our analyses to women who resided within close proximity to stations measuring CO and not PM_10_. One explanation for this may be that CO concentrations in general tended to be higher at CO-only stations. For example, the 75th, 90th, and 95th percentiles for third-trimester CO averages based on CO-only stations at the ZIP-code level were 2.02, 2.87, and 3.52 ppm, respectively whereas for the stations measuring CO and PM_10_ these values were 1.70, 2.14, and 2.43 ppm, respectively. We examined the composition of the populations around both types of monitoring stations with respect to individual maternal characteristics such as age, race/ethnicity, and education, and no clear pattern distinguishing them emerged. Furthermore, we used U.S. Census data for the year 2000 (U.S. Census Bureau 2004) to look at factors such as percent living in poverty (based on block groups within 2 mi of a station) and ethnic composition and found no differences between the two types of stations except that two of the CO-only stations were located in wealthier areas.

Another possible explanation is that CO may be a better marker of traffic emissions in the geographic areas surrounding CO-only stations versus areas surrounding stations that measure both CO and PM_10_ and that some unmeasured component in traffic exhaust is in fact responsible for the observed effects attributed to CO in our models. We tried to assess this by examining correlations between station-specific distance-weighted traffic density (DWTD) values and pollutant concentrations measured at each station for the year 2000. A DWTD measure was derived for each station using methods described in our previous study (Wilhelm and Ritz 2003). Year 2000 annual average daily traffic counts on streets within 2,000 feet from each station were weighted by the distance from the station to the street using a Gaussian probability distribution. We accounted for the influence of wind direction on the dispersion of exhaust from roadways by incorporating the percentage of time each station was annually downwind of a street into the DWTD value. Correlations between DWTD and annual average concentrations of CO and NO_2_, pollutants typically considered indicative of traffic exhaust, were positive at CO-only stations (*r* = 0.54 for CO, *r* = 0.55 for NO_2_) compared with small and negative correlations seen for stations measuring both CO and PM_10_ (*r* = –0.17 for CO, *r* = –0.32 for NO_2_). Interestingly, annual average O_3_ was negatively correlated with DWTD at CO-only stations (*r* = –0.91) but not at CO+PM_10_ stations (*r* = 0.16). O_3_ is a secondary pollutant formed through photochemical atmospheric reactions, and NO released directly in motor vehicle exhaust scavenges O_3_ to form NO_2_. Therefore, the negative correlation between O_3_ and traffic density at CO-only stations may reflect the greater contribution of motor vehicle emissions to air pollution in these areas. These correlations for the 12 LA County monitoring stations (Figure 2) suggest that CO may be a better marker of traffic exhaust exposure (although still imperfect) in the areas surrounding the CO-only stations; thus, the associations we observed for women residing in the vicinity of these stations may in fact be due to some unmeasured traffic exhaust component. Additional toxicologic and monitoring data are needed to investigate this hypothesis further.

The most important source of bias in this study is exposure misclassification. We discussed the sources of this misclassification at length in previous reports (Ritz and Yu 1999; Ritz et al. 2000; Wilhelm and Ritz 2003). Restricting our analyses to women who lived in close proximity to a station (within 1 mi) increased our effect estimates. Assuming that the misclassification inherent in our analyses is nondifferential, our results suggest that CO and particulate concentrations at an ambient monitoring station are better predictors of actual exposure for subjects living in close proximity to the station. This held true for pollutants that are usually considered to have relatively homogeneous spatial distributions over larger areas, such as PM_10_ and PM_2.5_. Hypothesizing that the observed effects are due to specific traffic exhaust pollutants for which CO and particles are mere proxies, it seems that ambient monitoring stations do not adequately capture the effects of primary exhaust pollutants expected to be more heterogeneously distributed throughout neighborhoods, such that ambient monitors misrepresent exposures beyond a 1-mi radius. Thus, our new results confirmed our suspicions that nondifferential exposure misclassification would generally increase and effect estimates decrease if local heterogeneity was important and that effects would not be adequately captured for homes at greater distances from monitoring stations.

Another potential source of bias in this study is residual confounding due to risk factors we were unable to account for in our analyses (e.g., maternal stature and weight gain during pregnancy, active and passive tobacco smoke exposure, stress). We recently completed a survey of approximately 2,500 LA County women who gave birth during 2003 to collect information on such factors. Therefore, in future analyses we will be able to assess directly whether these factors are an important source of bias in our analyses. The survey also included information on residential and occupational history, amount of commuting, and exposure to indoor air pollution sources during pregnancy. In the future, we will be able to examine more closely the importance of these factors for our air pollution results.

## Conclusions

As in our previous studies, we observed associations between elevated concentrations of CO and PM_10_ both early and late in pregnancy and risk of term LBW and preterm birth for women residing in the SoCAB and giving birth between 1994 and 2000. Thus, our previous results were generally confirmed for CO and PM_10_, even though concentrations of these two pollutants decreased in the SoCAB throughout the 1990s. We also observed some-what smaller effects for CO and PM_10_ averaged over the entire pregnancy period and risk of term LBW, similar to some previous reports in the literature. Restricting our analyses to women who lived within close proximity of monitoring stations appeared to reduce exposure misclassification and effect attenuation. Effects also were greater for women residing near stations measuring CO and not PM_10_, and we propose that this occurs because CO might be a better marker of traffic emissions in these LA locations. Improved exposure assessment methods may help to reduce misclassification and pinpoint important air pollution sources. Additional toxicologic or mechanistic studies may help shed more light on the effects observed in epidemiologic studies.

## CORRECTION

In the section “Preterm birth” and in Table 5, several of the values were incorrect in the manuscript originally published online. They have been corrected here.

## Figures and Tables

**Table 1:** Incidence of term LBW and preterm births by demographic characteristics: ZIP-code–level cohort.^a^

	Term LBW			Preterm		
Parameter	No. of births or mean ± SD	No. of cases or mean ± SD	Incidence (95% CI)	No. of births or mean ± SD	No. of cases or mean ± SD	Incidence (95% CI)
Mean gestational age (days)	275.5 ± 16.3	273.5 ± 10.8		276.0 ± 15.6	241.9 ± 20.3	
Mean birth weight (g)	3366.1 ± 542.3	2255.2 ± 276.3		3363.3 ± 505.5	2865.58 ± 727.5	
LBW (< 2,500 g)	136,134	2,778	2.0 (2.0–2.1)	4,382	2,400	54.8 (53.3–56.2)
Preterm (< 37 weeks)	—	—	—	106,483	9,268	8.7 (8.5–8.9)
Infant sex						
Male	70,015	1,188	1.7 (1.6–1.8)	54,086	5,022	9.3 (9.0–9.5)
Female	66,018	1,590	2.4 (2.3–2.5)	52,397	4,246	8.1 (7.9–8.3)
Prenatal care						
None	919	35	3.8 (2.6–5.0)	774	179	23.1 (20.2–26.1)
During first trimester	110,662	2,174	2.0 (1.9–2.0)	85,810	6,929	8.1 (7.9–8.3)
After first trimester	23,793	555	2.3 (2.1–2.5)	19,315	2,063	10.7 (10.2–11.1)
Parity						
First birth	51,831	1,275	2.5 (2.3–2.6)	39,795	3,546	8.9 (8.6–9.2)
Second or subsequent birth	84,303	1,503	1.8 (1.7–1.9)	66,688	5,722	8.6 (8.4–8.8)
Time since previous live birth						
≤12 months	2,199	57	2.6 (1.9–3.3)	1,833	328	17.9 (16.1–19.6)
> 12 months	132,862	2,686	2.0 (1.9–2.1)	103,788	8,842	8.5 (8.3–8.7)
Maternal race/ethnicity						
White	25,418	374	1.5 (1.3–1.6)	19,330	1,365	7.1 (6.7–7.4)
Hispanic	86,285	1,652	1.9 (1.8–2.0)	68,587	5,964	8.7 (8.5–8.9)
African American	11,624	426	3.7 (3.3–4.0)	8,572	1,110	12.9 (12.2–13.7)
Asian	7,687	182	2.4 (2.0–2.7)	6,138	451	7.3 (6.7–8.0)
Other	4,783	136	2.8 (2.4–3.3)	3,604	361	10.0 (9.0–11.0)
Maternal education (years)						
< 9	25,766	470	1.8 (1.7–2.0)	20,547	1,884	9.2 (8.8–9.6)
9–11	32,103	765	2.4 (2.2–2.5)	25,812	2,454	9.5 (9.1–9.9)
12	37,885	830	2.2 (2.0–2.3)	29,487	2,615	8.9 (8.5–9.2)
13–15	21,604	410	1.9 (1.7–2.1)	16,416	1,311	8.0 (7.6–8.4)
≥16	17,658	277	1.6 (1.4–1.8)	13,328	895	6.7 (6.3–7.1)
Maternal age (years)						
< 20	16,688	458	2.7 (2.5–3.0)	14,156	1,551	11.0 (10.4–10.5)
20–29	72,912	1,418	1.9 (1.8–2.0)	58,602	4,742	8.1 (7.9–8.3)
30–34	29,386	524	1.8 (1.6–1.9)	21,998	1,858	8.4 (8.1–8.8)
35–39	13,961	277	2.0 (1.8–2.2)	9,692	895	9.2 (8.7–9.8)
≥40	3,169	100	3.2 (2.5–3.8)	2,019	219	10.8 (9.5–12.2)
Previous LBW or preterm infant						
One or more	1,426	92	6.5 (5.2–7.7)	783	150	19.2 (16.4–21.9)
None	134,708	2,686	2.0 (1.9–2.1)	105,700	9,118	8.6 (8.5–8.8)
Birth season						
Winter	32,781	602	1.8 (1.7–2.0)	25,567	2,356	9.2 (8.9–9.6)
Spring	35,594	735	2.1 (1.9–2.2)	28,001	2,298	8.2 (7.9–8.5)
Summer	34,468	716	2.1 (1.9–2.2)	26,908	2,372	8.8 (8.5–9.2)
Fall	33,291	725	2.2 (2.0–2.3)	26,007	2,242	8.6 (8.3–9.0)

a Multiple births were excluded from the data set for term LBW (cohort size = 136,134); multiple births and births by cesarean section were excluded from the data set for preterm birth (cohort size = 106,483).

**Table 2:** Incidence of term LBW and preterm births by air pollution exposure: ZIP-code–level cohort.^a^

Parameter	No. of births	No. of cases	Incidence (95% CI)
Term LBW: third trimester			
Percentile of average CO exposure (ppm)^b^			
< 0.91	32,510	604	1.9 (1.7–2.0)
0.91 to < 1.82	65,212	1,323	2.0 (1.9–2.1)
≥1.82	32,366	755	2.3 (2.2–2.5)
Percentile of average PM_10_ exposure (μg/m^3^)			
< 32.8	19,805	404	2.0 (1.8–2.2)
32.8 to < 43.4	39,351	798	2.0 (1.9–2.1)
≥43.4	19,912	435	2.2 (2.0–2.4)
Percentile of average PM_2.5_ exposure (μg/m^3^)			
< 17.1	5,593	134	2.4 (2.0–2.8)
17.1 to < 24.0	11,209	250	2.2 (2.0–2.5)
≥24.0	5,988	124	2.1 (1.7–2.4)
Percentile of average O_3_ exposure (pphm)			
< 1.38	33,733	785	2.3 (2.2–2.5)
1.38 to < 2.87	66,990	1,329	2.0 (1.9–2.1)
≥2.87	33,814	643	1.9 (1.8–2.0)
Percentile of average NO_2_ exposure (pphm)			
< 3.02	32,442	615	1.9 (1.7–2.0)
3.02 to < 4.40	64,308	1,334	2.1 (2.0–2.2)
≥4.40	32,207	712	2.2 (2.1–2.4)
Preterm birth: first trimester			
Percentile of average CO exposure (ppm)			
< 0.97	25,499	2,212	8.7 (8.3–9.0)
0.97 to < 1.87	51,206	4,371	8.5 (8.3–8.8)
≥1.87	25,427	2,335	9.2 (8.8–9.5)
Percentile of average PM_10_ exposure (μg/m^3^)			
< 32.9	15,662	1,364	8.7 (8.3–9.2)
32.9 to < 43.9	31,388	2,758	8.8 (8.5–9.1)
≥43.9	15,793	1,353	8.6 (8.1–9.0)
Percentile of average PM_2.5_ exposure (μg/m^3^)			
< 18.0	3,262	347	10.6 (9.6–11.7)
18.0 to < 25.4	6,352	560	8.8 (8.1–9.5)
≥25.4	3,416	309	9.0 (8.1–10.0)
Percentile of average O_3_ exposure (pphm)			
< 1.36	26,461	2,338	8.8 (8.5–9.2)
1.36 to < 2.85	52,694	4,654	8.8 (8.6–9.1)
≥2.85	26,562	2,222	8.4 (8.0–8.7)
Percentile of average NO_2_ exposure (pphm)			
< 3.05	25,434	2,183	8.6 (8.2–8.9)
3.05 to < 4.42	50,515	4,442	8.8 (8.5–9.0)
≥4.42	25,279	2,267	9.0 (8.6–9.3)
Preterm birth: 6 weeks before birth			
Percentile of average CO exposure (ppm)			
< 0.87	25,498	2,176	8.5 (8.2–8.9)
0.87 to < 1.82	50,964	4,353	8.5 (8.3–8.8)
≥1.82	25,466	2,350	9.2 (8.9–9.6)
Percentile of average PM_10_ exposure (μg/m^3^)			
< 31.8	15,564	1,373	8.8 (8.4–9.3)
31.8 to < 44.1	31,121	2,686	8.6 (8.3–8.9)
≥44.1	15,722	1,383	8.8 (8.4–9.2)
Percentile of average PM_2.5_ exposure (μg/m^3^)			
< 16.5	4,305	355	8.2 (7.4–9.1)
16.5 to < 24.7	8,257	726	8.8 (8.2–9.4)
≥24.7	4,378	420	9.6 (8.7–10.5)
Percentile of average O_3_ exposure (pphm)			
< 1.29	26,299	2,338	8.9 (8.5–9.2)
1.29 to < 2.92	52,527	4,455	8.5 (8.2–8.7)
≥2.92	26,341	2,361	9.0 (8.6–9.3)
Percentile of average NO_2_ exposure (pphm)			
< 2.96	25,236	2,232	8.8 (8.5–9.2)
2.96 to < 4.41	50,359	4,380	8.7 (8.5–8.9)
≥4.41	25,183	2,227	8.8 (8.5–9.2)

a Multiple births were excluded from the data set for term LBW (cohort size = 136,134); multiple births and births by cesarean section were excluded from the data set for preterm birth (cohort size = 106,483).

b Values listed are the < 25th, 25 to < 75th, and ≥75th percentiles.

**Table 3:** Pollutant averages (ranges) and Pearson correlation coefficients for all pollutants by pregnancy period: ZIP-code–level cohort.^a^

		Pearson correlation coefficients			
Trimester/pollutant	Mean (range)	CO	NO_2_	O_3_	PM_10_
First trimester					
CO (ppm)	1.42 (0.26–2.82)	1.0			
NO_2_ (pphm)	3.91 (2.06–6.20)	0.81	1.0		
O_3_ (pphm)	2.15 (0.43–4.12)	−0.31	−0.47	1.0	
PM_10_ (μg/m^3^)	42.2 (26.3–77.4)	0.12	0.29	−0.01	1.0
PM_2.5_ (μg/m^3^)	21.9 (11.8–38.9)	0.57	0.73	–0.55	0.43
Third trimester					
CO (ppm)	1.21 (0.23–2.93)	1.0			
NO_2_ (pphm)	3.73 (2.01–6.24)	0.84	1.0		
O_3_ (pphm)	2.22 (0.38–4.18)	−0.36	−0.51	1.0	
PM_10_ (μg/m^3^)	41.5 (25.7–74.6)	0.32	0.45	−0.08	1.0
PM_2.5_ (μg/m^3^)	21.0 (11.8–38.9)	0.67	0.78	−0.60	0.52
Six weeks before birth					
CO (ppm)	1.42 (0.02–5.88)	1.0			
NO_2_ (pphm)	3.70 (0.76–7.46)	0.83	1.0		
O_3_ (pphm)	2.11 (0.15–5.85)	−0.37	−0.53	1.0	
PM_10_ (μg/m^3^)	39.1 (13.0–103.7)	0.36	0.49	−0.16	1.0
PM_2.5_ (μg/m^3^)	21.0 (9.9–48.5)	0.63	0.74	−0.60	0.60

a Pollutant averages and correlation coefficients are based on the entire data set (i.e., singleton term LBW births, singleton, vaginal preterm births, and controls) for all averaging periods except for the third trimester in which preterm births were excluded.

**Table 4:** Results for singleton term LBW [ORs (95% CIs) (*n* = cases, noncases)]: third trimester.

	CO				PM_10_	
Measure	Single-pollutant model	Multipollutant model (CO, NO_2_, O_3_) a	Multipollutant model (CO, NO_2_, O_3_, PM_10_) a	Measure	Single-pollutant model	Multipollutant model (CO, NO_2_, O_3_, PM_10_) a
Distance ≤1 mi^b^	(*n* = 653, 28,144)	(*n* = 628, 27,352)	(*n* = 221, 10,160)	Distance ≤1 mi	(*n* = 247, 10,981)	(*n* = 221, 10,160)
Per 1 ppm	1.10 (0.98–1.23)	1.15 (0.98–1.35)	1.21 (0.85–1.74)	Per 10 μg/m^3^	1.22 (1.05–1.41)	1.36 (1.12–1.65)
0.96 to < 1.84^c^	1.08 (0.88–1.33)	1.07 (0.83–1.38)	1.10 (0.72–1.69)	33.4 to < 44.4	1.08 (0.76–1.52)	1.16 (0.77–1.74)
≥1.84	1.36 (1.04–1.76)	1.29 (0.92–1.81)	1.39 (0.77–2.49)	≥44.4	1.48 (1.00–2.19)	1.58 (0.95–2.62)
1 < distance ≤2 mi	(*n* = 2,077, 87,049)	(*n* = 2,058, 85,847)	(*n* = 873, 39,497)	1 < distance ≤2 mi	(*n* = 895, 40,803)	(*n* = 873, 39,497)
Per 1 ppm	1.05 (0.99–1.13)	1.03 (0.94–1.13)	0.91 (0.76–1.10)	Per 10 μg/m^3^	0.98 (0.90–1.06)	1.02 (0.92–1.14)
0.95 to < 1.83	1.05 (0.94–1.18)	1.03 (0.90–1.17)	1.05 (0.86–1.29)	33.4 to < 44.7	0.95 (0.80–1.13)	0.93 (0.77–1.12)
≥1.83	1.10 (0.95–1.28)	1.07 (0.89–1.28)	0.97 (0.73–1.30)	≥44.7	0.96 (0.78–1.18)	1.02 (0.79–1.32)
2 < distance ≤4 mi	(*n* = 6,888, 293,904)	(*n* = 6,857, 292,020)	(*n* = 3,378, 143,981)	2 < distance ≤4 mi	(*n* = 3,424, 146,347)	(*n* = 3,378, 143,981)
Per 1 ppm	1.06 (1.02–1.10)	1.04 (0.99–1.10)	1.01 (0.92–1.11)	Per 10 μg/m^3^	1.03 (0.99–1.08)	1.04 (0.98–1.09)
0.96 to < 1.85	1.06 (1.00–1.13)	1.04 (0.96–1.11)	1.08 (0.98–1.20)	33.9 to < 45.0	1.04 (0.96–1.14)	1.02 (0.92–1.12)
≥1.85	1.08 (1.00–1.18)	1.05 (0.95–1.17)	1.11 (0.96–1.29)	≥45.0	1.08 (0.97–1.20)	1.06 (0.93–1.21)
ZIP-code level: SoCAB^d^	(*n* = 2,596, 112,495)	(*n* = 2,487, 107,053)	(*n* = 1,473, 62,604)	ZIP-code level: SoCAB	(*n* = 1,592, 68,652)	(*n* = 1,473, 62,604)
Per 1 ppm	1.12 (1.05–1.19)	1.10 (1.01–1.21)	0.99 (0.85–1.15)	Per 10 μg/m^3^	1.03 (0.97–1.09)	1.07 (0.99–1.15)
0.90 to < 1.75	1.13 (1.02–1.25)	1.12 (0.99–1.28)	1.02 (0.86–1.20)	33.2 to < 43.6	0.98 (0.86–1.11)	0.97 (0.85–1.12)
≥1.75	1.28 (1.12–1.47)	1.29 (1.08–1.53)	0.97 (0.78–1.22)	≥43.6	1.03 (0.88–1.21)	1.09 (0.90–1.31)

a For multipollutant model continuous results, all pollutants are entered as continuous variables; for multipollutant model categorical results, all pollutants are entered as categorical variables using the following percentiles of the concentration distributions: < 25th (reference group), 25th to 75th, ≥75th.

b The address-level analyses included the following LA County stations: Azusa, Burbank, Long Beach, Reseda, Pomona, Lynwood, Central LA, Pasadena, Hawthorne, West LA, Pico Rivera, and Santa Clarita.

c Values listed are the 25 to < 75th, and ≥ 75th percentiles.

d Includes ZIP codes that fell ≥60% by area within a 2-mi radius of the following stations: Azusa, Burbank, Long Beach, Reseda, Pomona, Lynwood, Central LA, Pasadena, Hawthorne, West LA, Anaheim, La Habra, El Toro/Lake Forest (after 1999 becomes Mission Viejo), Costa Mesa, Upland, and San Bernardino. The following variables were included in the models: infant sex, maternal age, race/ethnicity, and education, interval since previous live birth, previous LBW or preterm infant, level of prenatal care, birth season, parity, gestational age, and gestational age squared.

**Table 5:** Results for singleton, vaginally-delivered preterm births—RRs (95% CIs) (*n* = cases, noncases).^a^

	CO				PM_10_			PM_2.5_
Measure	Single-pollutant model	Multipollutant model (CO, NO_2_, O_3_) b	Multipollutant model (CO, NO_2_, O_3_, PM_10_) b	Measure	Single-pollutant model	Multipollutant model (CO, NO_2_, O_3_, PM_10_) b	Measure	Single-pollutant model
1st Trimester				1st Trimester			1st Trimester	
Distance ≤1 mi^c^	(*n* = 2,073, 21,931)	(*n* = 2,018, 21,277)	(*n* = 735, 7,948)	Distance ≤1 mi	(*n* = 792, 8,622)	(*n* = 735, 7,948)	Distance ≤1 mi	(*n* = 291, 2,701)
Per 1 ppm	1.06 (1.00–1.12)	1.10 (1.01–1.20)	0.99 (0.83–1.18)	Per 10 μg/m^3^	1.00 (0.93–1.09)	1.00 (0.90–1.12)	Per 10 μg/m^3^	0.85 (0.70–1.02)
1.05 to < 1.92^d^	1.00 (0.91–1.11)	1.05 (0.93–1.18)	0.96 (0.78–1.17)	33.3 to < 45.1	1.07 (0.90–1.26)	1.12 (0.92–1.36)	18.1 to < 25.2	0.91 (0.72–1.16)
≥1.92	1.18 (1.03–1.34)	1.27 (1.07–1.50)	1.03 (0.78–1.36)	≥45.1	1.12 (0.91–1.38)	1.17 (0.90–1.50)	≥25.2	0.83 (0.60–1.14)
1 < distance ≤2 mi	(*n* = 6,662, 68,100)	(*n* = 6,599, 67,236)	(*n* = 2,997, 31,419)	1 < distance ≤2 mi	(*n* = 3,067, 32,351)	(*n* = 2,997, 31,419)	1 < distance ≤2 mi	(*n* = 913, 8,763)
Per 1 ppm	1.06 (1.03–1.10)	1.04 (0.99–1.09)	0.94 (0.86–1.03)	Per 10 μg/m^3^	1.01 (0.97–1.05)	1.04 (0.99–1.10)	Per 10 μg/m^3^	0.85 (0.74–0.99)
1.03 to < 1.90	0.95 (0.90–1.01)	0.90 (0.84–0.96)	0.92 (0.83–1.01)	33.7 to < 45.3	1.03 (0.95–1.12)	1.07 (0.98–1.17)	18.3 to < 25.2	0.81 (0.69–0.94)
≥1.90	1.09 (1.01–1.17)	0.98 (0.90–1.08)	0.99 (0.86–1.14)	≥45.3	1.07 (0.97–1.19)	1.13 (1.00–1.27)	≥25.2	0.79 (0.65–0.97)
2 < distance ≤4 mi	(*n* = 24,339, 229,969)	(*n* = 24,274, 228,586)	(*n* = 12,205, 113,902)	2 < distance ≤4 mi	(*n* = 12,311, 115,594)	(*n* = 12,205, 113,902)	2 < distance ≤4 mi	(*n* = 4,025, 35,222)
Per 1 ppm	1.08 (1.06–1.09)	1.05 (1.02–1.07)	1.05 (1.01–1.10)	Per 10 μg/m^3^	1.01 (0.99–1.03)	0.99 (0.97–1.02)	Per 10 μg/m^3^	0.83 (0.78–0.88)
1.05 to < 1.90	0.98 (0.95–1.01)	0.93 (0.90–0.96)	0.94 (0.89–0.99)	34.1 to < 45.5	1.03 (0.99–1.08)	0.99 (0.95–1.04)	18.5 to < 24.9	0.79 (0.74–0.85)
≥1.90	1.11 (1.07–1.16)	1.03 (0.99–1.08)	1.06 (1.00–1.14)	≥45.5	1.02 (0.96–1.07)	0.94 (0.89–1.01)	≥24.9	0.76 (0.70–0.84)
ZIP-code level: SoCAB^e^	(*n* = 8,592, 88,869)	(*n* = 8,244, 84,473)	(*n* = 4,916, 50,087)	ZIP-code level: SoCAB	(*n* = 5,304, 54,888)	(*n* = 4,916, 50,087)	ZIP-code level: SoCAB	(*n* = 1,059, 9,895)
Per 1 ppm	1.04 (1.01–1.07)	1.03 (0.98–1.08)	0.97 (0.90–1.04)	Per 10 μg/m^3^	0.99 (0.96–1.01)	0.99 (0.96–1.03)	Per 10 μg/m^3^	0.73 (0.67–0.80)
0.95 to < 1.81	0.97 (0.93–1.02)	0.95 (0.90–1.02)	0.92 (0.85–0.99)	33.3 to < 44.2	1.01 (0.95–1.08)	1.03 (0.97–1.11)	18.0 to < 25.4	0.70 (0.61–0.80)
≥1.81	1.05 (0.99–1.12)	1.01 (0.93–1.10)	0.95 (0.85–1.06)	≥44.2	0.98 (0.90–1.05)	1.01 (0.92–1.11)	≥25.4	0.64 (0.53–0.76)
Six weeks before birth				Six weeks before birth			Six weeks before birth	
Distance ≤1 mi^c^	(*n* = 2,074, 21,930)	(*n* = 2,017, 21,294)	(*n* = 734, 7,964)	Distance ≤1 mi	(*n* = 792, 8,608)	(*n* = 734, 7,964)	Distance ≤1 mi	(*n* = 378, 3,778)
Per 1 ppm	1.04 (0.98–1.09)	1.10 (1.03–1.18)	0.98 (0.83–1.16)	Per 10 μg/m^3^	1.02 (0.95–1.10)	1.06 (0.97–1.16)	Per 10 μg/m^3^	1.09 (0.91–1.30)
0.92 to < 1.84^d^	1.00 (0.91–1.11)	1.00 (0.89–1.12)	0.96 (0.77–1.19)	32.5 to < 44.8	1.09 (0.92–1.29)	1.09 (0.90–1.31)	16.8 to < 24.1	1.21 (0.97–1.51)
≥1.84	1.01 (0.89–1.15)	1.01 (0.85–1.18)	0.85 (0.62–1.15)	≥44.8	1.12 (0.92–1.37)	1.17 (0.91–1.49)	≥24.1	1.25 (0.93–1.68)
1 < distance ≤2 mi	(*n* = 6,662, 68,054)	(*n* = 6,589, 67,147)	(*n* = 2,987, 31,325)	1 < distance ≤2 mi	(*n* = 3,066, 32,293)	(*n* = 2,987, 31,325)	1 < distance ≤2 mi	(*n* = 1,185, 12,170)
Per 1 ppm	1.04 (1.01–1.08)	1.10 (1.05–1.14)	1.01 (0.93–1.09)	Per 10 μg/m^3^	1.00 (0.96–1.03)	1.01 (0.97–1.06)	Per 10 μg/m^3^	1.08 (0.97–1.21)
0.91 to < 1.85	1.04 (0.98–1.10)	1.08 (1.01–1.15)	0.97 (0.88–1.08)	32.3 to < 45.3	0.99 (0.91–1.07)	1.00 (0.92–1.10)	17.2 to < 24.5	0.94 (0.82–1.08)
≥1.85	1.14 (1.06–1.22)	1.22 (1.11–1.33)	0.97 (0.84–1.11)	≥45.3	0.99 (0.89–1.10)	1.02 (0.91–1.16)	≥24.5	1.04 (0.87–1.24)
2 < distance ≤4 mi	(*n* = 24,313, 229,724)	(*n* = 24,244, 228,335)	(*n* = 12,175, 113,642)	2 < distance ≤4 mi	(*n* = 12,282, 115,326)	(*n* = 12,175, 113,642)	2 < distance ≤4 mi	(*n* = 5,229, 48,855)
Per 1 ppm	1.01 (0.99–1.02)	1.03 (1.00–1.05)	1.03 (0.99–1.08)	Per 10 μg/m^3^	0.99 (0.98–1.01)	1.00 (0.98–1.02)	Per 10 μg/m^3^	1.05 (0.99–1.10)
0.93 to < 1.87	1.02 (0.99–1.05)	1.02 (0.99–1.06)	0.98 (0.94–1.04)	33.1 to < 45.6	1.00 (0.96–1.05)	1.01 (0.96–1.05)	17.3 to < 24.6	1.06 (1.00–1.13)
≥1.87	1.04 (1.00–1.08)	1.05 (1.00–1.10)	1.00 (0.94–1.08)	≥45.6	0.98 (0.93–1.03)	0.98 (0.92–1.04)	≥24.6	1.08 (0.99–1.17)
ZIP-code level: SoCAB^e^	(*n* = 8,589, 89,039)	(*n* = 8,252, 84,678)	(*n* = 4,898, 50,048)	ZIP-code level: SoCAB	(*n* = 5,285, 54,721)	(*n* = 4,898, 50,048)	ZIP-code level: SoCAB	(*n* = 1,381, 14,047)
Per 1 ppm	1.03 (1.00–1.06)	1.08 (1.04–1.13)	0.99 (0.92–1.06)	Per 10 μg/m^3^	1.02 (0.99–1.04)	1.02 (0.99–1.06)	Per 10 μg/m^3^	1.10 (1.00–1.21)
0.87 to < 1.75	1.00 (0.95–1.05)	1.01 (0.95–1.07)	0.96 (0.89–1.04)	32.1 to < 44.3	1.01 (0.95–1.07)	1.02 (0.95–1.09)	16.5 to < 24.7	1.06 (0.94–1.20)
≥1.75	1.04 (0.98–1.11)	1.09 (1.00–1.18)	0.94 (0.84–1.05)	≥44.3	1.04 (0.96–1.12)	1.04 (0.95–1.14)	≥24.7	1.19 (1.02–1.40)

a ORs were adjusted to RRs.

b For multipollutant model continuous results, all pollutants are entered as continuous variables; for multipollutant model categorical results, all pollutants are entered as categorical variables using the following percentiles of the concentration distributions: < 25th (reference group), 25th to 75th, ≥75th.

c The address-level analyses included the following LA County stations: Azusa, Burbank, Long Beach, Reseda, Pomona, Lynwood, Central LA, Pasadena, Hawthorne, West LA, Pico Rivera, and Santa Clarita.

d Values listed are the 25th to < 75th, and ≥ 75th percentiles.

e Includes ZIP codes that fell ≥60% by area within a 2-mi radius of the following stations: Azusa, Burbank, Long Beach, Reseda, Pomona, Lynwood, Central LA, Pasadena, Hawthorne, West LA, Anaheim, La Habra, El Toro/Lake Forest (after 1999 becomes Mission Viejo), Costa Mesa, Upland, and San Bernardino. The following variables were included in the models: infant sex, maternal age, race/ethnicity, and education, interval since previous live birth, previous LBW or preterm infant, level of prenatal care, birth season, and parity.
